# Anti-DFS70 Antibodies for Differentiating Systemic Autoimmune Rheumatic Disease in Patients with Positive ANA Tests: A Systematic Review and Meta-Analysis

**DOI:** 10.3390/diagnostics11091592

**Published:** 2021-09-01

**Authors:** Chiao-Feng Cheng, Ming-Chieh Shih, Ting-Yuan Lan, Ko-Jen Li

**Affiliations:** 1 Department of Internal Medicine, National Taiwan University Hospital Yun-Lin Branch, Yun-Lin County 640, Taiwan; chiaofengcheng@gmail.com; 2 Institute of Epidemiology and Preventive Medicine, College of Public Health, National Taiwan University, Taipei City 100, Taiwan; littlecanargie@gmail.com; 3 Department of Internal Medicine, National Taiwan University Hospital Hsin-Chu Branch, Hsin-Chu City 300, Taiwan; d20023579@gmail.com; 4 Department of Internal Medicine, National Taiwan University Hospital, Taipei City 100, Taiwan

**Keywords:** ANA-associated rheumatic disease, systemic autoimmune rheumatic disease, dense fine speckled

## Abstract

Anti-DFS70 antibodies have been proposed as a marker to exclude systemic autoimmune rheumatic disease (SARD). We conducted this systematic diagnostic test accuracy review and meta-analysis to determine the performance of anti-DFS70 antibodies in patients with a positive anti-nuclear antibody (ANA) test result to exclude SARD. We searched PubMed, Embase, Web of Science, Scopus and the Cochrane Library up to 22 February 2021, and included studies examining the diagnostic accuracy of anti-DFS70 antibodies in patients with a positive ANA test result. The results were pooled using a hierarchical bivariate model and plotted in summary receiver operating characteristic curves. R software and Stata Statistical Software were used for the statistical analysis. Eight studies with 4168 patients were included. The summary sensitivity was 0.19 (95% confidence interval: 0.12–0.28) and the specificity was 0.93 (95% confidence interval: 0.88–0.96). The area under the curve was 0.69 (95% confidence interval: 0.64–0.72). The meta-regression analysis showed that targeting only ANA-associated rheumatic disease was associated with higher specificity. In addition, the studies with a non-SARD prevalence of <80% and using a chemiluminescence assay were associated with higher specificity. Anti-DFS70 antibodies have high specificity for the exclusion of SARD among patients presenting with a positive ANA test, but the sensitivity is low.

## 1. Introduction

Anti-nuclear antibodies (ANAs) have a crucial role in the diagnosis of systemic autoimmune rheumatic disease (SARD). However, the high false positive rate of ANAs in healthy populations and in patients with non-autoimmune diseases may result in unnecessary anxiety and pose a burden on healthcare systems [1,2,3,4]. Anti-DFS70 antibodies cause a dense fine speckled (DFS) pattern in ANA tests [1,5,6,7]. Previous studies have shown that anti-DFS70 antibodies are commonly found in the serum of healthy people [1,8,9], and that therefore, in contrast to other autoantibodies associated with specific autoimmune diseases, anti-DFS70 antibodies may not be associated with SARD. Several studies have shown that without other common anti-extractable nuclear antigen (anti-ENA) antibodies, anti-DFS70 antibodies rarely exist in SARD patients [2,10,11,12,13]. Therefore, monospecific anti-DFS70 antibodies, defined as the presence of anti-DFS70 antibodies without other common anti-ENA antibodies, are regarded as a reliable marker to exclude SARD [14].

However, a recent meta-analysis focusing on the diagnostic performance of anti-DFS70 antibodies among patients presenting with a DFS pattern in an ANA test found substantial heterogeneity in both the sensitivity and specificity [15]. The difficulty in recognizing the DFS pattern [16,17,18] and the presence of other autoantibodies that produce a DFS pattern in ANA tests [19,20] may not only contribute to the heterogeneity but also hinder the application of the study results to clinical practice. In contrast, although heterogeneity still exists, the identification of a positive ANA test may be more widely adopted and validated in laboratories worldwide than a DFS pattern [16,17,18]. Therefore, focusing on studies enrolling patients presenting with a positive ANA test, rather than a DFS pattern, for meta-analysis may decrease the heterogeneity between studies and increase the applicability of their results. Accordingly, we conducted this systematic diagnostic test accuracy review and meta-analysis to determine the performance of anti-DFS70 antibodies in excluding SARD for patients presenting with a positive ANA test.

## 2. Materials and Methods

We registered this systematic review and meta-analysis protocol on PROSPERO (PROSPERO ID: CRD42021238714). We followed the Preferred Reporting Items for Systematic Reviews and Meta-Analysis Diagnostic Test Accuracy (PRISMA-DTA) guidelines throughout the literature search process to structure and design the framework for the review [21].

### 2.1. Literature Search

A comprehensive literature search was undertaken to identify all of the published studies reporting the diagnostic power of anti-DFS70 antibodies to exclude SARD. The following electronic databases were searched from inception to 22 February 2021: PubMed, EMBASE, Web of Science, Scopus and the Cochrane Library (accessed on 22 February 2021). Data S1 in the Supplementary Material details the search strings used for each database. Additional studies were identified through a manual search of the bibliographies in the included studies and relevant narrative reviews.

### 2.2. Selection Criteria

Studies investigating the detection of anti-DFS70 antibodies in patients were selected for full-text review. The inclusion criteria were as follows: (1) studies evaluating the presence of anti-DFS70 antibodies in patients with a positive ANA test, or an equivalent test; and (2) studies in which the clinical diagnoses of SARD and non-SARD were confirmed. The exclusion criteria were as follows: (1) insufficient data to determine the diagnostic accuracy; (2) case-control-type accuracy studies; (3) a shared study population with other studies; (4) studies in which no cases of SARD were reported; and (5) studies with fewer than 10 patients. We excluded case-control-type studies due to the high risk of introducing bias in the evaluation of the diagnostic test accuracy. Conference abstracts and letters were eligible if sufficient information was available from the report. Literature and conference abstracts in all languages were evaluated. We emailed the authors to request unpublished data or to clarify the study method as needed. Two reviewers (C.F.C. and T.Y.L.) systematically and independently performed the title/abstract screening, followed by a full-text review to ensure quality and accuracy throughout the process. Any disagreements regarding the inclusion or exclusion of studies were resolved by discussion. If disagreements were still present after the discussion, a third reviewer (K.J.L.) was consulted. During the data extraction and quality assessment, any disagreements were managed by the same process.

### 2.3. Data Extraction

Two reviewers (C.F.C. and T.Y.L.) systematically and independently performed the data extraction. The following data were extracted into an electronic table and assessed by C.F.C. and T.Y.L.: the first author’s name, year of publication, number of patients, ANA titer at enrollment in the study, method of detecting anti-DFS70 antibodies, reference standard for SARD, and number of true positive (TP), false negative (FN), true negative (TN) and false positive (FP) participants. A TP was defined as a positive anti-DFS70 antibody test result in non-SARD patients. A FN was defined as a negative test result in non-SARD patients. A TN was defined as a negative test result in SARD patients. A FP was defined as a positive test result in SARD patients. Because there is currently no consensus on the definition of SARD, the definition of SARD in the enrolled studies varied. Specifically, some studies reported the diagnostic performance of anti-DFS70 antibodies for only ANA-associated rheumatic disease (AARD) rather than all types of SARD, including systemic lupus erythematosus (SLE), Sjögren’s syndrome, systemic sclerosis, idiopathic inflammatory myositis (IIM) and mixed connective tissue disease (MCTD). A meta-regression analysis was performed to explore the impact of different definitions of SARD.

### 2.4. Quality Assessment

Two reviewers (C.F.C. and T.Y.L.) assessed the risk of bias and quality for each study individually at the study level. The Quality Assessment of Diagnostic Accuracy Studies revised version 2 (QUADAS-2) was used for the quality assessment [22]. The tool is comprised of four domains: patient selection, index test, reference standard, and flow and timing. Each domain was assessed and reported as a high, low, or unclear risk of bias and applicability.

### 2.5. Statistical Analysis

We aggregated the accuracy of the diagnostic tests by creating a 2 × 2 table of TP, FN, TN and FP rates for each study based on the extracted data. We plotted the sensitivities and specificities with their 95% confidence intervals (CIs) in coupled forest plots. For the meta-analysis of the diagnostic accuracy measures, we used the hierarchical bivariate modeling framework [23] to estimate the summary operating points (i.e., a summary value for sensitivity and specificity) and the 95% confidence regions around the summary operating points. We also plotted summary receiver operating characteristic curves. The area under the curve (AUC) for the test was also calculated, and a 95% confidence interval was estimated [24]. Probability-modifying plots based on the summary estimates of the sensitivity and specificity of the tests were used to visualize the post-test probability of non-SARD [25]. We assessed heterogeneity by examining forest plots of sensitivity and specificity across the studies for variability in the study estimates and the overlap of 95% CIs. We used a linear mixed model to perform meta-regression analysis for relevant clinical parameters to explore heterogeneity [26]. In order to compare the difference in heterogeneity in the enrolled studies between this study and previous meta-analysis focusing on patients with a DFS pattern [15], we used tau-squared (τ^2^) to estimate the between-studies variance of the sensitivities and specificities in the enrolled studies. A large τ^2^ denotes a large between-study variance in the sensitivities or specificities in the enrolled studies. In addition, we used a 95% prediction interval to describe the distribution of true values of the sensitivity and specificity 95% of the time. We assessed the publication bias using funnel plots and Deeks’ test [27]. A *p*-value < 0.1 in Deeks’ test was taken to suggest the presence of publication bias. We performed all of the analyses using R software version 3.6.3 (R Foundation for Statistical Computing, Vienna, Austria) [28] with the meta [29] and mada [26] packages, and Stata Statistical Software: Release 14 (StataCorp. 2015. College Station, TX, USA) with the midas program [24].

## 3. Results

**Figure 1:**
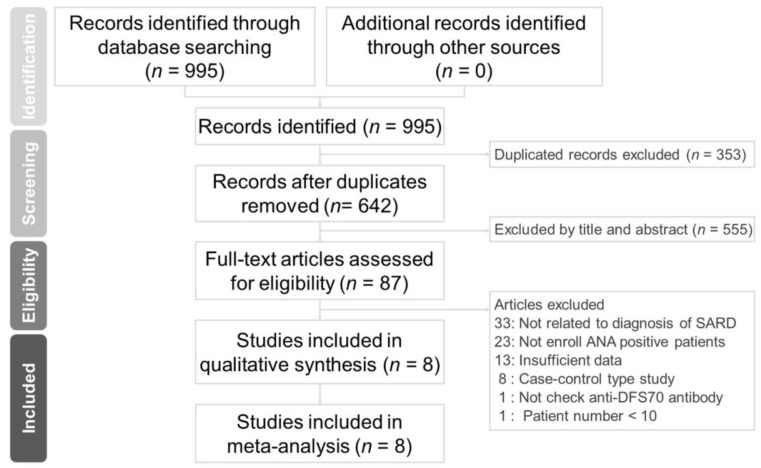
Flowchart of the study selection for the current meta-analysis.

The electronic search identified 642 records after removing duplicates. Among these 642 records, the full texts of 87 were assessed. The study flow is summarized in Figure 1. Eventually, eight studies were included both in the quantitative and qualitative analyses, with a total of 4168 participants (from 102 [30] to 1968 [31]).

### 3.1. Quality Assessment

**Figure 2:**
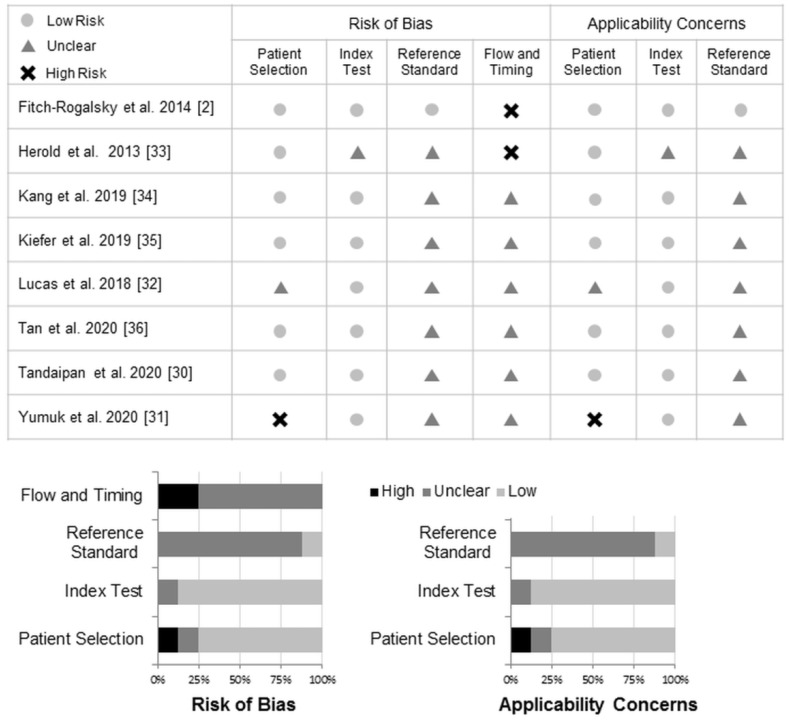
Methodological quality of the included studies.

Figure 2 shows the results of the qualitative evaluation. In the patient selection domains, one study was assessed as having a high risk of bias and concerns of applicability because it only enrolled patients with available results of ANA and anti-ENA [31]. One study was assessed as having an unclear risk due to inadequate information [32]. For the index test, one study was assessed as having an unclear risk of bias and concerns of applicability because the index test threshold was not reported [33]. For the reference standard, only one of the studies applied the classification criteria of SARD [2]. Due to possible heterogeneity in the clinical diagnosis and practice, the studies using reference standards other than classification criteria were assessed as having an unclear risk of bias and applicability in the reference standard [30,31,32,33,34,35,36]. For flow and timing, because none of the enrolled studies specified the time interval between the index test and the diagnosis of SARD, the appropriateness of the interval between the index test (anti-DFS70 antibodies) and the reference standard (the diagnosis of SARD) could not be assessed. Therefore, the risk of bias in the flow and timing was assessed as being unclear in six studies. In the other two studies, because some of the enrolled patients were not included in the final analysis due to missing information on the clinical diagnosis, the risk of bias for flow and timing was assessed as being high [2,33].

### 3.2. Main Characteristics of the Included Studies

A variety of Hep-2 methods were used for the ANA test (Table 1). The ANA titer for inclusion in these studies varied from 1:40 to 1:160. With regards to the methods of anti-DFS70 antibody detection, four studies used a chemiluminescence assay (CIA), two used a line immunoassay (LIA), one used an enzyme-linked immunosorbent assay, and one used a Western blot. The prevalence of non-SARD varied from 51.7% to 90.5%. Only one study used classification criteria for the diagnosis of SARD, and the other studies used clinical diagnosis, diagnosis from medical records, or did not specify the method of diagnosis.

### 3.3. The Diagnostic Performance of Anti-DFS70 Antibodies in the Exclusion of SARD

**Figure 3:**
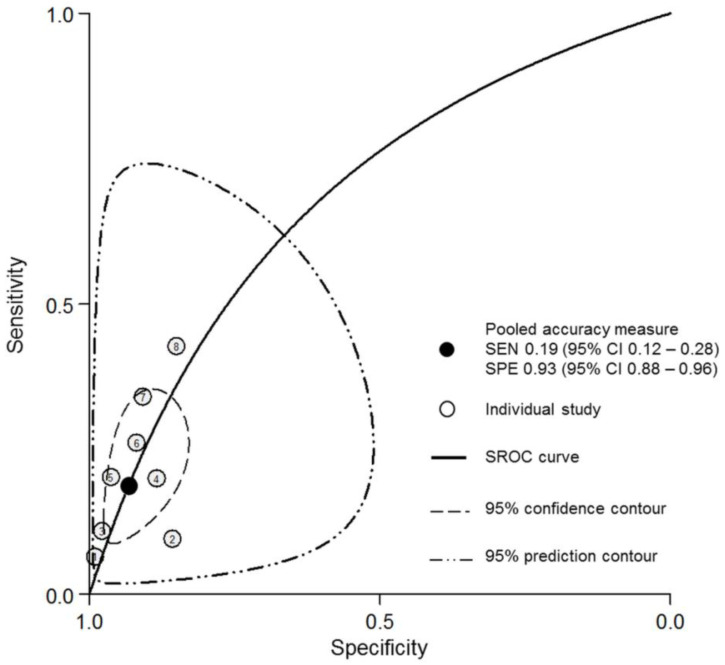
SROC curve for anti-DFS70 antibodies to exclude SARD in patients with a positive ANA test result. ANA, anti-nuclear antibody; SARD, systemic autoimmune rheumatic disease; SEN, sensitivity; SPE, specificity; SROC curve, summary receiver operating characteristic curve.

Figure 3 shows that anti-DFS70 antibodies had a pooled sensitivity of 0.19 (95% CI: 0.12–0.28) and a pooled specificity of 0.93 (95% CI: 0.88–0.96) to exclude SARD in patients with a positive ANA test. The AUC was 0.69 (95% CI: 0.64–0.72). In the probability modifying plot, the positive likelihood ratio was 2.76 (95% CI: 1.61–4.73), and the negative likelihood ratio was 0.87 (95% CI: 0.8–0.95) (Figure S1).

### 3.4. Heterogeneity

**Figure 4:**
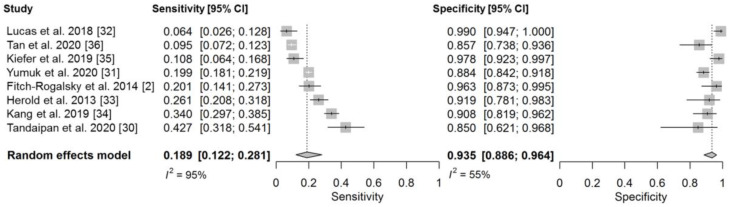
Paired forest plot for the sensitivity and specificity of anti-DFS70 antibodies in patients with a positive ANA test result.

The paired forest plots showed substantial heterogeneity among the studies, as is common with meta-analyses of diagnostic accuracy studies (Figure 4). Both the sensitivity and specificity estimates varied widely; however, heterogeneity was more evident in the sensitivity than the specificity. Compared with a previous meta-analysis focusing on patients with a DFS pattern [15], the τ^2^ was smaller for both sensitivity and specificity in the present study (Figure S2). The prediction intervals were also narrower for both sensitivity and specificity in the present study than in the previous meta-analysis [15]. The meta-regression analysis for the exploration of the causes of the heterogeneity showed that the articles limiting the targeted diseases to only AARD had higher specificity than those targeting all types of SARD (Table S1). In addition, the studies with a non-SARD prevalence of <80% were associated with higher specificity than those with a non-SARD prevalence of ≥80%. The studies using the CIA method to detect anti-DFS70 antibodies also had higher specificity than those using other methods. The article type did not obviously impact the diagnostic performance of anti-DFS70 antibodies.

### 3.5. Publication Bias

A visual assessment of the funnel plot of the anti-DFS70 antibodies (Figure S3) did not show significant asymmetry. The Deeks’ test was not statistically significant either (*p* = 0.27). However, this finding did not exclude publication bias because Deeks’ test lacks power, particularly in the presence of high heterogeneity.

## 4. Discussion

In this systematic review and meta-analysis, we found that in patients with a positive ANA test, anti-DFS70 antibodies had a high specificity of 0.93 (95% CI: 0.88–0.96) to exclude SARD, but a low sensitivity of 0.19 (95% CI: 0.12–0.28). For the exclusion of AARD, the specificity of anti-DFS70 antibodies was higher than for the targeting of SARD.

The anti-DFS70 antibody targets DFS70 protein and appears as a DFS pattern in an ANA test [6]. The DFS70 protein upregulates the expression of anti-oxidant, stress response and cancer-associated genes in various cell types, and is regarded as a stress activated transcription co-activator [37]. However, the physiological function and importance of DFS70 protein in non-disease conditions remain largely unknown [37]. Despite being found in some inflammatory diseases, such as atopic diseases and eye diseases initially [3], the anti-DFS70 antibody also occurs commonly in sera from patients with non-SARD conditions, and even in healthy subjects (up to 21.6%) [3,4,6]. This finding lead to the subsequent studies exploring the potential role of the anti-DFS70 antibody as a marker to “exclude SARD” [3,7,8,9]. Mahler et al. further proposed that the anti-DFS70 antibody may reflect the background B cell autoantibody repertoire [4].

Although most experts suggest using monospecific anti-DFS70 antibodies to exclude SARD [2,13,14], we found that in patients presenting with a positive ANA test, using anti-DFS70 antibodies alone without concomitant autoantibody tests could still achieve good specificity [38]. In apparently healthy individuals, the prevalence of anti-DFS70 antibodies has been reported to be no higher than 30% [3]. Similarly, we found that anti-DFS70 antibodies were present in 3.8–37.3% of individuals with a positive ANA test, and in 6.4–42.7% of those with non-SARD and a positive ANA test. These findings suggest that although anti-DFS70 antibodies may be common in individuals with non-SARD and a positive ANA test, the prevalence may not be high enough to achieve good sensitivity for the detection of non-SARD.

In this study, we also found that different definitions of SARD may influence the diagnostic performance of anti-DFS70 antibodies. In the literature, the term SARD describes autoimmune disorders affecting multiple organs, such as SLE, systemic sclerosis, rheumatoid arthritis and systemic vasculitis [31,36,39]. However, a positive ANA test is only essential in the diagnosis of some disorders included in SARD [40]. In recent years, to specify the diseases which rely on ANAs for a diagnosis, some experts have suggested the term “AARD”, which includes only SLE, Sjögren’s syndrome, systemic sclerosis, IIM and MCTD [2,41]. In the present study, anti-DFS70 antibodies had higher specificity in the exclusion of AARD than all types of SARD. Therefore, we suggest using anti-DFS70 antibodies to exclude only AARD among patients presenting with a positive ANA test.

We also found that a higher prevalence of non-SARD was associated with a lower specificity of anti-DFS70 antibodies. The diagnostic performance of a test has been reported to potentially vary according to the prevalence of the disease [42,43]. Leefang et al. found that, in every one of three meta-analyses of diagnostic test accuracy, there was a significant association between disease prevalence and sensitivity or specificity [43]. Currently, the change in disease prevalence is not considered to be a single cause of the change in specificity or sensitivity [42], and factors that could affect both disease prevalence and diagnostic accuracy may be a more plausible explanation [42]. However, the actual mechanisms and causes still remain to be clarified [43].

Another interesting finding of this study is that the detection of anti-DFS70 antibodies using the CIA method had a higher specificity than other methods. Bonroy et al. reported that the CIA method tended to detect anti-DFS70 antibodies in samples without other common anti-ENA antibodies; however, the mechanism remains unclear [44]. Because the presence of anti-DFS70 antibodies without other anti-ENA antibodies (“monospecific” anti-DFS70) is more specific for the exclusion of SARD [14,15], the results reported by Bonroy et al. may explain why the CIA method had higher specificity than the other methods in our study. Further studies are needed to verify this finding and explore the cause of this phenomenon.

This study has several limitations. First, only two enrolled studies reported the diagnostic performance of monospecific anti-DFS70 antibodies to exclude SARD. The small number of studies precluded the further analysis of the performance of monospecific anti-DFS70 antibodies. However, considering the low sensitivity of anti-DFS70 antibodies to detect non-SARD, the added value of monospecific anti-DFS70 antibody tests seems to be limited, given their higher specificity but lower sensitivity compared to anti-DFS70 antibodies alone [15]. Second, the interpretation of ANA tests can be influenced by many factors. In the laboratory, the definition of a positive threshold for dilution, the type of assay used to detect ANAs, and whether the definition of a positive result includes cytoplasmic and mitotic patterns are all possible confounding factors. However, this information was not comprehensively reported in the included studies, limiting the further analysis of their impact on diagnostic performance in the present study. Third, the number of healthy subjects included in our analysis may be limited. Considering that the sera available for analysis in most studies usually came from daily clinical practice, it is reasonable to assume that the subjects tested for ANA were likely to have some initial symptoms suggestive of SARD. This might limit the application of the study results to health examination or screening for the general population.

In conclusion, in this study we found that anti-DFS70 antibodies had high specificity to exclude SARD among patients presenting with a positive ANA test. Regarding the relatively low sensitivity, testing the anti-DFS70 antibody in patients with a DFS pattern, rather than every patient with a positive ANA, may improve the efficiency of the diagnostic procedure. In order to optimize the specificity, we suggest using anti-DFS70 antibodies to exclude only SLE, Sjögren’s syndrome, IIM, systemic sclerosis and MCTD.

## Figures and Tables

**Table 1:** Main characteristics of the included studies.

Authors	Country	ANA Detection Method	Titer	Anti-DFS70 Method	SARD Diagnosis	Non-SARD Prevalence	TP	FP	FN	TN
Fitch-Rogalsky et al., 2014 [2]	Canada	Hep-2000 (ImmunoConcepts)	1:160	CIA (Inova)	Classification criteria ^1^	74.0%	31	2	123	52
Herold et al., 2013 [33]	Austria	Not specified	Not specified	CIA (Inova)	Medical record ^1^	87.6%	68	3	193	34
Kang et al., 2019 [34]	South Korea	Kallestad HEp-2 Kit (Bio-Rad)	1:40	Western blot	Not specified ^2^	86.0%	158	7	307	69
Kiefer et al., 2019 [35]	Germany	Not specified	1:80	CIA (Inova)	Clinical diagnosis ^1^	63.3%	17	2	140	89
Lucas et al., 2018 [32]	New Zealand	HEp-2 I.I.F. (Inova) or EIA ANA Screening Test (Bio-Rad)	Not specified	CIA (Inova)	Not specified ^2^	51.7%	7	1	102	101
Tan et al., 2020 [36]	Singapore	Hep 2010 (Euroimmun)	1:80	ELISA (Euroimmun)	Medical record ^3^	90.5%	51	8	485	48
Tandaipan et al., 2020 [30]	Spain	Not specified	1:80	LIA (Euroimmun)	Not specified ^2^	80.4%	35	3	47	17
Yumuk et al., 2020 [31]	Turkey	HEp 2010 (Euroimmun)	1:100	LIA (Euroimmun)	Medical record ^4^	85.1%	334	34	1341	259

^1^ The definition of SARD was limited to ANA-associated rheumatic diseases, including systemic lupus erythematosus, Sjögren’s syndrome, dermatomyositis/polymyositis, systemic sclerosis, mixed connective tissue disease, and overlap syndrome. ^2^ The definition of SARD was not specified. ^3^ The definition of SARD included systemic lupus erythematosus, Sjögren’s syndrome, idiopathic inflammatory myositis, systemic sclerosis, mixed connective tissue disease, undifferentiated connective tissue disease, and systemic vasculitis. ^4^ The definition of SARD included systemic lupus erythematosus, Sjögren’s syndrome, idiopathic inflammatory myositis, systemic sclerosis, and rheumatoid arthritis. ANA, anti-nuclear antibody; CIA, chemiluminescence assay; DFS, dense fine speckled; ELISA, enzyme-linked immunosorbent assay; FN, false negative; FP, false positive; LIA, line immunoassay; SARD, systemic autoimmune rheumatic disease; TN, true negative; TP, true positive.

## Data Availability

The data presented in this study are available within the article and in the Supplementary Materials.

## Supplementary Materials

The following are available online at https://www.mdpi.com/article/10.3390/diagnostics11091592/s1, Data S1: Search Strategy; Figure S1: Probability modifying plot of anti-DFS70 antibodies to exclude SARD; Figure S2: Comparison of the heterogeneity between the studies with a positive ANA and studies with a DFS pattern; Figure S3: Deeks’ funnel plot asymmetry test for publication bias; Table S1: Results of the meta-regression analysis of the diagnostic accuracy of anti-DFS70 antibodies.

## References

[1] Mariz H.A., Sato E.I., Barbosa S.H., Rodrigues S.H., Dellavance A., Andrade L.E. (2011). Pattern on the antinuclear antibody-HEp-2 test is a critical parameter for discriminating antinuclear antibody-positive healthy individuals and patients with autoimmune rheumatic diseases. Arthritis Rheum..

[2] Fitch-Rogalsky C., Steber W., Mahler M., Lupton T., Martin L., Barr S.G., Mosher D.P., Wick J., Fritzler M.J. (2014). Clinical and serological features of patients referred through a rheumatology triage system because of positive antinuclear antibodies. PLoS ONE.

[3] Conrad K., Rober N., Andrade L.E., Mahler M. (2017). The Clinical Relevance of Anti-DFS70 Autoantibodies. Clin. Rev. Allergy Immunol..

[4] Mahler M., Andrade L.E., Casiano C.A., Malyavantham K., Fritzler M.J. (2019). Anti-DFS70 antibodies: An update on our current understanding and their clinical usefulness. Expert Rev. Clin. Immunol..

[5] Ochs R.L., Stein T.W., Peebles C.L., Gittes R.F., Tan E.M. (1994). Autoantibodies in interstitial cystitis. J. Urol..

[6] Ochs R.L., Mura Y., Si Y., Ge H., Chan E.K.L., Mtan E. (2000). Autoantibodies to DFS 70 kd/transcription coactivator p75 in atopic dermatitis and other conditions. J. Allergy Clin. Immunol..

[7] Dellavance A., Viana V.S., Leon E.P., Bonfa E.S., Andrade L.E., Leser P.G. (2005). The clinical spectrum of antinuclear antibodies associated with the nuclear dense fine speckled immunofluorescence pattern. J. Rheumatol..

[8] Watanabe A., Kodera M., Sugiura K., Usuda T., Tan E.M., Takasaki Y., Tomita Y., Muro Y. (2004). Anti-DFS70 Antibodies in 597 Healthy Hospital Workers. Arthritis Rheum..

[9] Albesa R., Sachs U., Infantino M., Manfredi M., Benucci M., Baus Y., Lutterbeck S., Andrade L., Morris K., Friedenberg A. (2019). Increased prevalence of anti-DFS70 antibodies in young females: Experience from a large international multi-center study on blood donors. Clin. Chem. Lab. Med..

[10] Lee H., Kim Y., Han K., Oh E.J. (2016). Application of anti-DFS70 antibody and specific autoantibody test algorithms to patients with the dense fine speckled pattern on HEp-2 cells. Scand. J. Rheumatol..

[11] Kang S.Y., Lee W.I. (2009). Clinical significance of dense fine speckled pattern in anti-nuclear antibody test using indirect immunofluorescence method. Korean J. Lab. Med..

[12] Muro Y., Sugiura K., Morita Y., Tomita Y. (2008). High concomitance of disease marker autoantibodies in anti-DFS70/LEDGF autoantibody-positive patients with autoimmune rheumatic disease. Lupus.

[13] Infantino M., Pregnolato F., Bentow C., Mahler M., Benucci M., Li Gobbi F., Damiani A., Grossi V., Franceschini F., Bodio C. (2019). Only monospecific anti-DFS70 antibodies aid in the exclusion of antinuclear antibody associated rheumatic diseases: An Italian experience. Clin. Chem. Lab. Med..

[14] Damoiseaux J., Andrade L.E.C., Carballo O.G., Conrad K., Francescantonio P.L.C., Fritzler M.J., Garcia De La Torre I., Herold M., Klotz W., Cruvinel W.D.M. (2019). Clinical relevance of HEp-2 indirect immunofluorescent patterns: The International Consensus on ANA patterns (ICAP) perspective. Ann. Rheum. Dis..

[15] Cheng C.F., Lan T.Y., Shih M.C., Li K.J. (2020). Monospecific anti-DFS70 antibodies are moderately helpful in excluding ANA-associated rheumatic disease in patients presenting with a dense fine speckled pattern—A systematic review and meta-analysis of diagnostic test accuracy. Autoimmun. Rev..

[16] Bizzaro N., Tonutti E., Villalta D. (2011). Recognizing the dense fine speckled/lens epithelium-derived growth factor/p75 pattern on HEP-2 cells: Not an easy task! Comment on the article by Mariz et al. Arthritis Rheum..

[17] Bentow C., Fritzler M.J., Mummert E., Mahler M. (2016). Recognition of the dense fine speckled (DFS) pattern remains challenging: Results from an international internet-based survey. Auto Immun. Highlights.

[18] Zheng B., Wang Z., Mora R.A., Liu A., Li C., Liu D., Zhai F., Liu H., Gong H., Zhou J. (2020). Anti-DFS70 Antibodies Among Patient and Healthy Population Cohorts in China: Results From a Multicenter Training Program Showing Spontaneous Abortion and Pediatric Systemic Autoimmune Rheumatic Diseases Are Common in Anti-DFS70 Positive Patients. Front. Immunol..

[19] Bizzaro N., Tonutti E., Visentini D., Alessio M.G., Platzgummer S., Morozzi G., Antico A., Villalta D., Piller-Roner S., Vigevani E. (2007). Antibodies to the lens and cornea in anti-DFS70-positive subjects. Ann. N. Y. Acad. Sci..

[20] Basu A., Woods-Burnham L., Ortiz G., Rios-Colon L., Figueroa J., Albesa R., Andrade L.E., Mahler M., Casiano C.A. (2015). Specificity of antinuclear autoantibodies recognizing the dense fine speckled nuclear pattern: Preferential targeting of DFS70/LEDGFp75 over its interacting partner MeCP2. Clin. Immunol..

[21] McInnes M.D.F., Moher D., Thombs B.D., McGrath T.A., Bossuyt P.M., Clifford T., Cohen J.F., Deeks J.J., Gatsonis C., Hooft L. (2018). Preferred Reporting Items for a Systematic Review and Meta-analysis of Diagnostic Test Accuracy Studies: The PRISMA-DTA Statement. JAMA.

[22] Whiting P.F., Rutjes A.W., Westwood M.E., Mallett S., Deeks J.J., Reitsma J.B., Leeflang M.M., Sterne J.A., Bossuyt P.M. (2011). QUADAS-2: A revised tool for the quality assessment of diagnostic accuracy studies. Ann. Intern. Med..

[23] Reitsma J.B., Glas A.S., Rutjes A.W., Scholten R.J., Bossuyt P.M., Zwinderman A.H. (2005). Bivariate analysis of sensitivity and specificity produces informative summary measures in diagnostic reviews. J. Clin. Epidemiol..

[24] Dwamena B. MIDAS: Stata Module for Meta-Analytical Integration of Diagnostic Test Accuracy Studies. Statistical Software Components S456880, Boston College Department of Economics, Revised 5 February 2009. https://ideas.repec.org/c/boc/bocode/s456880.html.

[25] Whiting P.F., Sterne J.A., Westwood M.E., Bachmann L.M., Harbord R., Egger M., Deeks J.J. (2008). Graphical presentation of diagnostic information. BMC Med. Res. Methodol..

[26] Doebler P. (2019). Mada: Meta-Analysis of Diagnostic Accuracy. R Package Version 0.5.10. https://CRAN.R-project.org/package=mada.

[27] Deeks J.J., Macaskill P., Irwig L. (2005). The performance of tests of publication bias and other sample size effects in systematic reviews of diagnostic test accuracy was assessed. J. Clin. Epidemiol..

[28] R Core Team (2019). R: A Language and Environment for Statistical Computing.

[29] Balduzzi S., Rucker G., Schwarzer G. (2019). How to perform a meta-analysis with R: A practical tutorial. Evid. Based Ment. Health.

[30] Tandaipan Jaime J.L., Magallares B., Bernardez J., Riera Alonso E., Pujalte F., Martínez-Martínez L., Baucells A., Castellví I., Corominas H., Martinez Pardo S. (2020). Fri0594 use of anti-dfs70 antibodies in rheumatological patients with suspicion of systemic autoimmune disease. Ann. Rheum. Dis..

[31] Yumuk Z., Demir M. (2020). Clinical value of anti-DFS70 antibodies in a cohort of patients undergoing routine antinuclear antibodies testing. J. Immunol. Methods.

[32] Lucas S., Chang W.L., Merien F. (2018). Prevalence and clinical significance of Anti-DFS70 in antinuclear antibody (ANA)-positive patients undergoing routine ANA testing in a New Zealand public hospital. J. Rheumatol..

[33] Herold M., Klotz W. (2013). Antinuclear antibodies positive but no autoimmune disease. Arthritis Rheum..

[34] Kang S.Y., Lee W.I., Kim M.H., La Jeon Y. (2019). Clinical use of anti-DFS70 autoantibodies. Rheumatol. Int..

[35] Kiefer D., von Brunn M., Baraliakos X., Andreica I., Braun J. (2020). Clinical significance of determination of DFS70 antibodies to rule out connective tissue diseases. Z. Rheumatol..

[36] Tan T.C., Ng C.Y.L., Khai Pang L. (2020). The clinical utility of anti-DFS70 for identifying antinuclear antibody-positive patients without systemic autoimmune rheumatic disease. Singap. Med. J..

[37] Ortiz-Hernandez G.L., Sanchez-Hernandez E.S., Casiano C.A. (2020). Twenty years of research on the DFS70/LEDGF autoantibody-autoantigen system: Many lessons learned but still many questions. Auto Immun. Highlights.

[38] Damoiseaux J., Andrade L.E.C., Fritzler M.J., Herold M., Infantino M., Von Muhlen C. (2019). Response to Titre-specific positive predictive value of anti-nuclear antibody patterns’ by Vulsteke et al. Ann. Rheum. Dis..

[39] Mahler M., Fritzler M.J. (2010). Epitope specificity and significance in systemic autoimmune diseases. Ann. N. Y. Acad. Sci..

[40] Solomon D.H., Kavanaugh A.J., Schur P.H. (2002). Evidence-based guidelines for the use of immunologic tests: Antinuclear antibody testing. Arthritis Rheum..

[41] Damoiseaux J., von Mühlen C.A., Garcia-De La Torre I., Carballo O.G., de Melo Cruvinel W., Francescantonio P.L.C., Fritzler M.J., Herold M., Mimori T., Satoh M. (2016). International consensus on ANA patterns (ICAP): The bumpy road towards a consensus on reporting ANA results. Auto Immun. Highlights.

[42] Leeflang M.M., Bossuyt P.M., Irwig L. (2009). Diagnostic test accuracy may vary with prevalence: Implications for evidence-based diagnosis. J. Clin. Epidemiol..

[43] Leeflang M.M., Rutjes A.W., Reitsma J.B., Hooft L., Bossuyt P.M. (2013). Variation of a test’s sensitivity and specificity with disease prevalence. CMAJ.

[44] Bonroy C., Schouwers S., Berth M., Stubbe M., Piette Y., Hoffman I., Devreese K., Van Hoovels L. (2018). The importance of detecting anti-DFS70 in routine clinical practice: Comparison of different care settings. Clin. Chem. Lab. Med..

