# Supplementary material for: Anti-DFS70 Antibodies for Differentiating Systemic Autoimmune Rheumatic Disease in Patients with Positive ANA Tests: A Systematic Review and Meta-Analysis
Source: Diagnostics (Basel). 2021 Sep 1;11(9):1592. doi: 10.3390/diagnostics11091592 (PMC8468616; doi:10.3390/diagnostics11091592)
Supplement: Supplementary file 1 [file diagnostics-11-01592-s001.zip › diagnostics-1345784-supplementary.pdf]

Supplementary Information

Data S1. Search Strategy

Figure S1. Probability modifying plot of anti-DFS70 antibodies to exclude SARD

Figure S2. Comparison of heterogeneity between studies with positive ANA and studies with a DFS pattern

Figure S3. Deeks' funnel plot asymmetry test for publication bias

Table S1. Results of meta-regression analysis of the diagnostic accuracy of anti-DFS70 antibodies

Data S1. Search Strategy

The following electronic databases were searched from inception to February 2021: PubMed, EMBASE, Web of Science, Scopus, and Cochrane Library. Additional studies were identified through a manual search of bibliographies in the included studies and relevant narrative reviews.

Search strings used for each database:

[PubMed]
(autoimmune disease OR rheumatic disease OR systemic autoimmune rheumatic disease OR ANA associated rheumatic disease) AND (DFS70 OR dense fine speckled OR PSIP1 protein, human)

[Embase]
('autoimmune' OR 'rheumatic' OR 'ana associated rheumatic disease' OR 'systemic autoimmune rheumatic disease') AND ('dfs70' OR 'dense fine speckled' OR 'psip1 protein' OR 'dense fine speckled 70 antibody' OR 'ledgf protein' OR 'p75 protein' OR 'dense fine speckles 70 protein' OR 'pc4 and sfrs1 interacting protein 1')

[Web of Science]
('autoimmune' OR 'rheumatic' OR 'ana associated rheumatic disease' OR 'systemic autoimmune rheumatic disease') AND ('dfs70' OR 'dense fine speckled' OR 'psip1 protein' OR 'dense fine speckled 70 antibody' OR 'ledgf protein' OR 'p75 protein' OR 'dense fine speckles 70 protein' OR 'pc4 and sfrs1 interacting protein 1')

[Scopus]
ALL ("autoimmune" OR "rheumatic" OR "ana associated rheumatic disease" OR "systemic autoimmune rheumatic disease"
AND
"dfs70" OR "dense fine speckled" OR "psip1 protein" OR "ledgf protein" OR "p75 protein" OR "pc4 and sfrs1 interacting protein 1")

[Cochrane Library]
('autoimmune' OR 'rheumatic' OR 'ana associated rheumatic disease' OR 'systemic autoimmune rheumatic disease') AND ('dfs70' OR 'dense fine speckled' OR 'psip1 protein' OR 'dense fine speckled 70 antibody' OR 'ledgf protein' OR 'p75 protein' OR 'dense fine speckles 70 protein' OR 'pc4 and sfrs1 interacting protein 1')

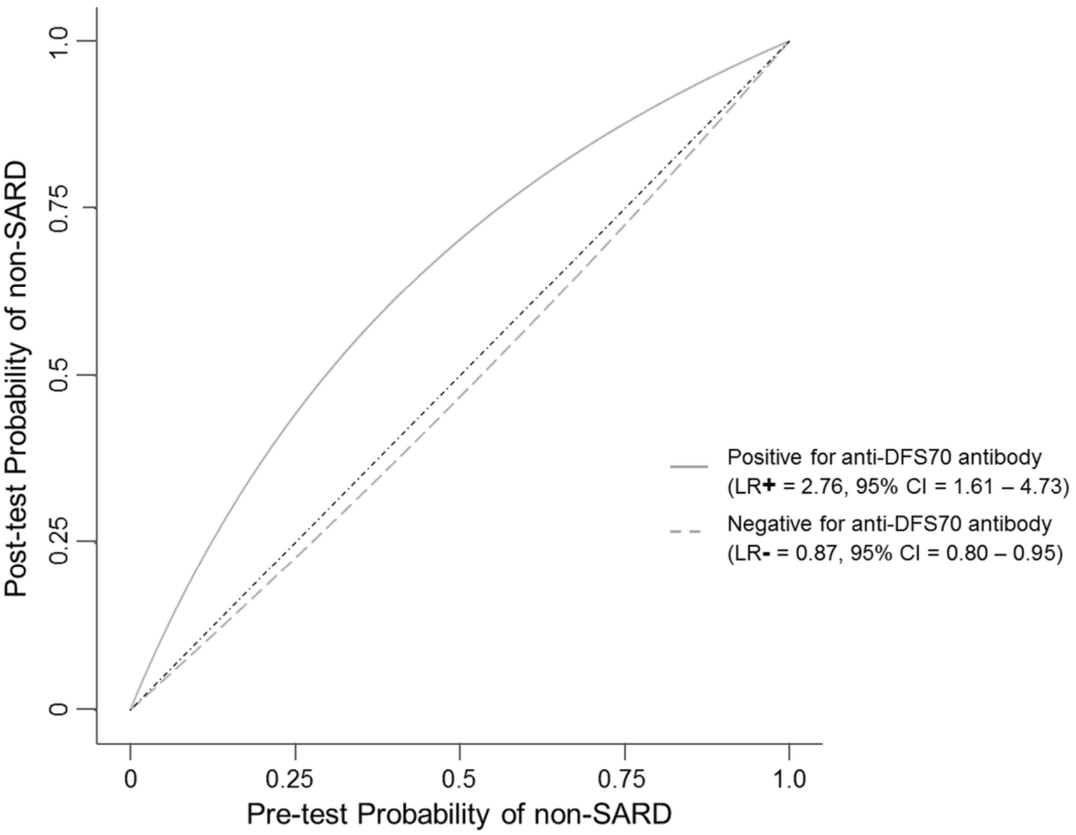

Figure S1. Probability modifying plot of anti-DFS70 antibodies to exclude SARD

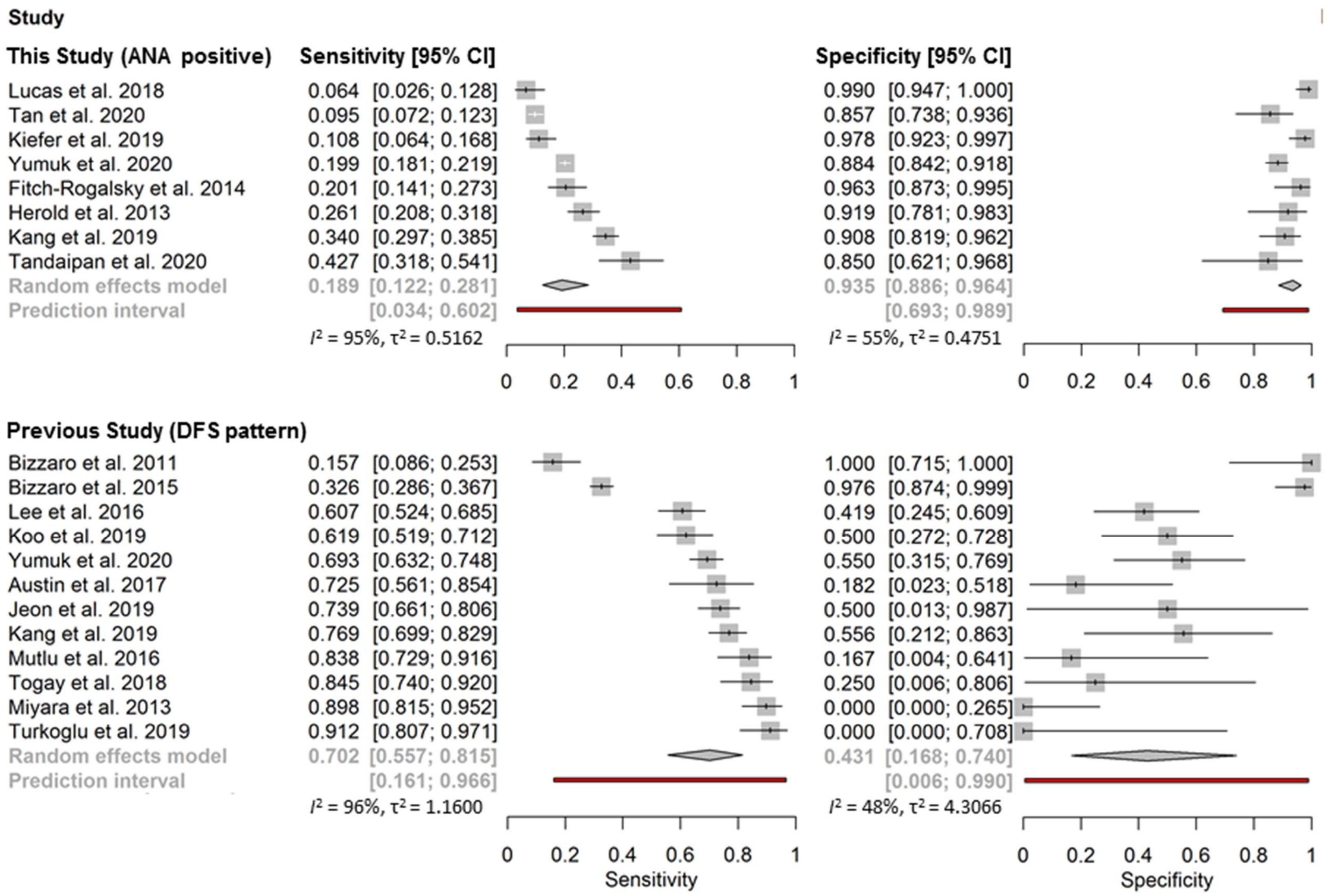

Figure S2. Comparison of heterogeneity between studies with positive ANA and studies with DFS pattern

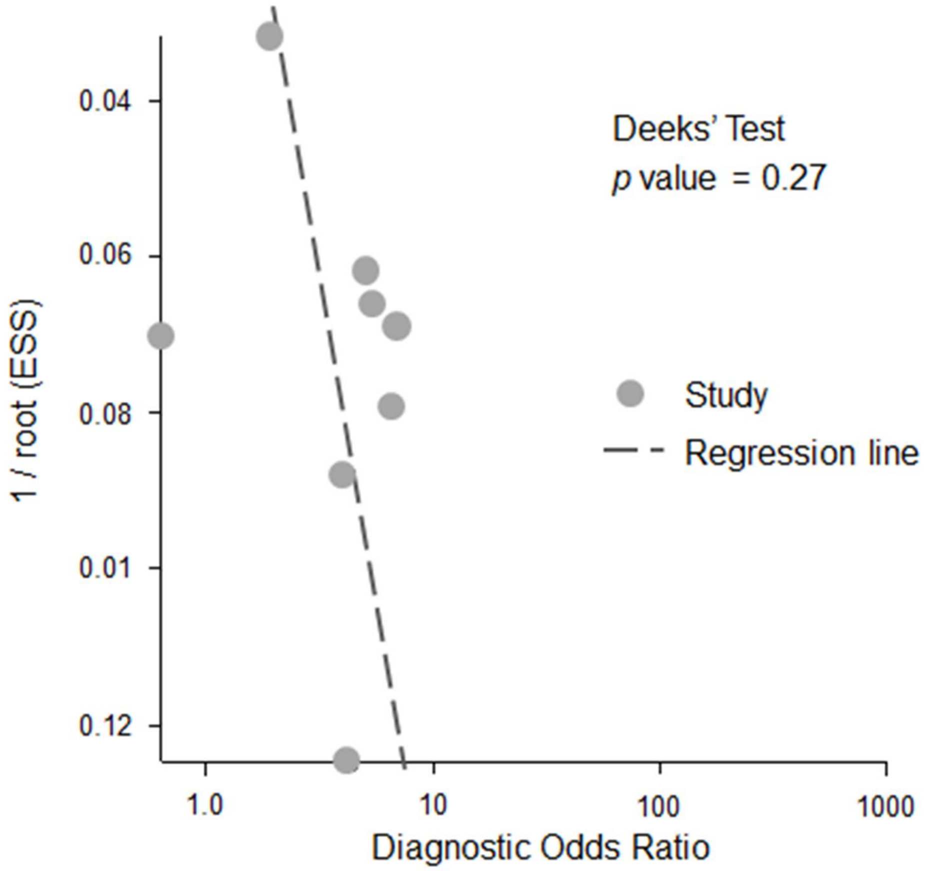

Figure S3. Deeks' funnel plot asymmetry test for publication bias

Table S1. Results of meta-regression analysis of the diagnostic accuracy of anti-DFS70 antibodies and monospecific anti-DFS70 antibodies

Covariate		Summary estimate for anti-DFS70 antibodies			
		Sensitivity (95% CI)	*p*-value	Specificity (95% CI)	*p*-value
Definition of SARD	Limited to AARD	0.18 (0.08 – 0.37)	-	0.96 (0.91 – 0.98)	-
	Not specified	0.19 (0.10 – 0.34)	0.913	0.89 (0.85 – 0.92)	0.021*
Non-SARD prevalence	Non-SARD < 80%	0.13 (0.05 – 0.24)	-	0.98 (0.95 – 0.99)	-
	Non-SARD ≥ 80%	0.24 (0.15 – 0.37)	0.100	0.89 (0.85 – 0.91)	< 0.001*
Anti-DFS70 method	CIA	0.15 (0.07 – 0.28)	-	0.96 (0.93 – 0.98)	-
	Others	0.24 (0.13 – 0.40)	0.283	0.88 (0.85 – 0.91)	0.001*
Article type	Article	0.17 (0.09 – 0.31)	-	0.92 (0.84 – 0.96)	
	Abstract/Letter	0.22 (0.09 – 0.42)	0.677	0.94 (0.82 – 0.98)	0.685

* p-value < 0.05

AARD, ANA-associated rheumatic disease; CI, confidence interval; CIA, chemiluminescence assay; SARD, systemic autoimmune rheumatic disease
